# Soft tagging of overlapping high confidence gene mention variants for cross-species full-text gene normalization

**DOI:** 10.1186/1471-2105-12-S8-S6

**Published:** 2011-10-03

**Authors:** Cheng-Ju Kuo, Maurice HT Ling, Chun-Nan Hsu

**Affiliations:** 1 Institute of Information Science, Academia Sinica, Taipei 115, Taiwan; 2 School of Chemical and Life Sciences, Singapore Polytechnic, Republic of Singapore; 3 Department of Zoology, The University of Melbourne, Parkville, Victoria, Australia; 4 Information Sciences Institute, University of Southern California, Marina del Rey, California, USA

## Abstract

**Background:**

Previously, gene normalization (GN) systems are mostly focused on disambiguation using contextual information. An effective gene mention tagger is deemed unnecessary because the subsequent steps will filter out false positives and high recall is sufficient. However, unlike similar tasks in the past BioCreative challenges, the BioCreative III GN task is particularly challenging because it is not species-specific. Required to process full-length articles, an ineffective gene mention tagger may produce a huge number of ambiguous false positives that overwhelm subsequent filtering steps while still missing many true positives.

**Results:**

We present our GN system participated in the BioCreative III GN task. Our system applies a typical 2-stage approach to GN but features a *soft tagging gene* mention tagger that generates a set of overlapping gene mention variants with a nearly perfect recall. The overlapping gene mention variants increase the chance of precise match in the dictionary and alleviate the need of disambiguation. Our GN system achieved a precision of 0.9 (F-score 0.63) on the BioCreative III GN test corpus with the silver annotation of 507 articles. Its TAP-*k* scores are competitive to the best results among all participants.

**Conclusions:**

We show that despite the lack of clever disambiguation in our gene normalization system, effective soft tagging of gene mention variants can indeed contribute to performance in cross-species and full-text gene normalization.

## Background

An essential step in biological text mining, gene normalization (GN) is to link a mention of gene or gene product appearing in a text to a standard database identifier referring to a specific gene entity [1,2]. Gene normalization is uniquely challenging for many reasons. First, genes are an evolving concept in biology and gene names are created and eliminated constantly. As a result, there are many nomenclature standards but authors rarely follow the standards strictly. Meanwhile, orthologous genes and gene products of different species may share the same names but have different database identifiers and create ambiguity [3].

Many GN systems have been developed since the BioCreative (BC) 1B task challenge evaluation [4]. The task is to normalize genes of three selected model organisms. The results suggest that GN performance may be organism-dependent due to the naming conventions associated with each organism [4]. The subsequent BioCreative II GN task focused only on human genes. The best team achieved a F-score of 0.81 [5], which is comparable to that of the BC 1B. A typical GN system usually consists of gene mention tagger, dictionary, matcher and disambiguation modules, a two-stage approach where the first stage is to use a tagger to tag all gene mentions in a text followed by the second stage of matching gene mentions to a dictionary and then resolving disambiguation, if multiple entries are matched [6]. Lau et al. [7] used a rule-based system and achieved a F-score of 0.76 whereas Hakenberg et al. [8] achieved an F-score of 0.86, also with a rule-based system. GENO [9] used both symbolic and statistical methods to achieve a F-score of 0.86 (precision: 0.878, recall: 0.850). All of the above results and many other related GN systems were evaluated on the BioCreative II GN dataset and applied a relatively simple or out-of-shelf gene mention tagger, and emphasized more on dictionary matching and disambiguation. Baumgartner et al. [10] and Vespoor et al. [11] therefore suggested that with clever dictionary matching and disambiguation, performance of a GN system is largely dependent on the recall but not the precision of the underlying gene mention tagger because false positive gene mentions will be filtered out. Since it is straightforward to have high recall if precision can suffer, this conclusion seems to implicitly imply that improvement in gene mention tagging is unnecessary for high performance GN systems. This point of view was further corroborated by the fact that some of the top performing gene mention taggers in the BioCreative II gene mention (GM) task failed to deliver good results in the BC II GN task, though this is partly because the training corpus of the BC II GM is incompatible with the BC II GN task. The GM corpus contains genes and gene products of all species as well as mentions of gene family that have no database IDs at all, while the BC II GN task focused only on human genes.

Unlike similar tasks in the past BioCreative challenges, the GN task of BioCreative III is particularly challenging because it is not species-specific. Moreover, instead of using abstracts, it is required to process full-length articles. These differences make the task closer to the real literature curation tasks by human curators but in such a setting, an ineffective gene mention tagger may produce a huge number of ambiguous false positives that overwhelm subsequent filtering steps while still missing many true positives of GN.

In this paper, we show that improvements in gene mention tagging can indeed contribute to performance in cross-species and full-text GN. We present our GN system participated in the BioCreative III GN task [12]. The gene mention tagger in our GN system is an advanced version of AIIAGMT (version 2.0.1) [13-15], which was first developed for the BioCreative II gene mention tagging (GM) task [16]. In 2007, we built a Conditional Random Fields (CRF)-based gene mention tagging system [17] by integrating bi-directional parsing with a rich set of features. It achieved a F-score of 0.8683 (with a precision of 0.8930 and a recall of 0.8449) on the BC II GM test corpus and ranked the second among 21 participants [17]. In order to maximize the potential of integrated bi-directional parsing models, we attempted different asymmetric feature settings to establish up to 6 models and integrated them according to the CRF output scores. The integrated model achieved 0.8830 in F-score (with precision 0.8895 and recall 0.8765), a significant improvement from the original version. This work was incorporated into the BioCreative MetaServer [15] and distributed independently as AIIAGMT [14] since.

We extended AIIAGMT further for the GN task. The latest version features soft tagging, in the sense that our tagger tags sets of overlapping gene mention variants with accurately estimated confidence scores. Soft tagging allows for a higher chance of precise match in the gene name dictionary. The recall of soft tagging is nearly perfect (0.9826 on the BioCreative II corpus) and therefore, it is highly likely that one of the overlapping variants is a correct gene mention. Soft tagging uses an ensemble of taggers for GN as in [10,14] but is fundamentally different in that soft tagging makes no attempt to select the best pair of boundaries for overlapping gene mention candidates but retains all high confident candidates for dictionary matching. Soft tagging effectively provides soft boundaries of a gene mention. As a result, the need to deal with spelling variations, partial matches and ambiguities can be alleviated.

Other key features of our GN system include:

• Short form-long form filtering: We have developed an accurate abbreviation definition detector BIOADI [18] and we used it to filter out incorrectly tagged gene mentions.

• Species resolution before dictionary matching: Since ambiguous gene names are rare within a single species [19], our system resolved species first and therefore minimized the need of disambiguation.

• Regular expression extension of Entrez Gene records retrieved by a document retrieval system: We applied a document retrieval system for dictionary matching. We have an idea of a data-driven approach to dictionary matching so that the problem of GN can be solved as information retrieval, but at this moment, we have not collected a sufficiently large corpus of documents to serve as the dictionary. Instead, we used regular expression to expand entries in Entrez Gene records and then used regular expression again to filter out false query results.

Our GN system achieved a precision of 0.64 and a recall of 0.59 (F-score of 0.614) on the BioCreative III GN training corpus and a precision of 0.90 and a recall of 0.49 (F-score of 0.633) on the BioCreative III GN test corpus with the silver annotation of 507 articles. Its TAP-*k* scores are competitive to the best results among all participants, never ranked below third in all combinations of test corpora and the selection of *k*.

## Methods

### Data preprocessing

The training and test data of the BioCreative III GN task, provided by the BioCreative organizers, consist of two file types for each example article: PubMed Central (PMC) XML files and PDF files. We parsed the PMC XML files to plain-text files by XPath. Then we extracted sections without a sec-type attribute to exclude References, Methods, Materials, etc. and segmented each remaining section into sentences as our input data for gene normalization. We did not use PDF files.

### System organization

**Figure 1:**
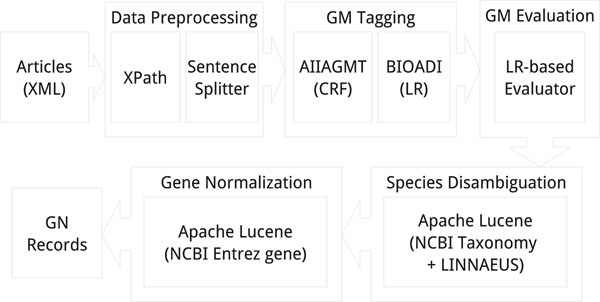
GN System Organization

Figure 1 illustrates the system organization of our GN system, which consists of four modules:

• gene mention tagging module (GMT) tags variants of overlapping gene or gene product mentions from the input text. These variants are candidate mentions that may overlap with other candidate mentions but have different boundaries.

• gene mention evaluation module (GME) evaluates the quality of gene mentions extracted by the GMT by estimating their confidence scores.

• species designation module (SD) designates a species (more specifically, taxonomy id) to each variant.

• gene normalization module (GNM) assigns a corresponding Entrez Gene record ID to each variant.

GMT and GME together accomplish the soft tagging of overlapping high confidence gene and gene product mention variants in our gene normalization system. The system was implemented in Java 1.6.0 running on 32-bit Linux. Implementation of model learning and inference algorithms that we used, including CRF and logistic regression, was modified from the source codes of MALLET 2.0 [20].

### Soft tagging: gene mention tagging module

Our gene mention tagger was modified from AIIAGMT that we developed [14]. One of the changes is that we reduced the number of features from ~5 million to ~2 million to improve the computational performance without sacrificing accuracy. The feature types removed were Part-of-Speech (POS) tags and sliding window features of POS-tags and case-sensitive words.

We then attempted to maximize the recall of our gene mention tagger as suggested in [6,10], yet maintain the false positives at a manageable level. We used a finding in our previous work that the union of the top most-probable tagging solutions by bi-directional parsing models may achieve a nearly perfect recall [14].

For example, given a sentence as follows:

Acetyl-CoA carboxylase from yeast is an essential enzyme and is regulated by factors that control phospholipid metabolism.

**Figure 2:**
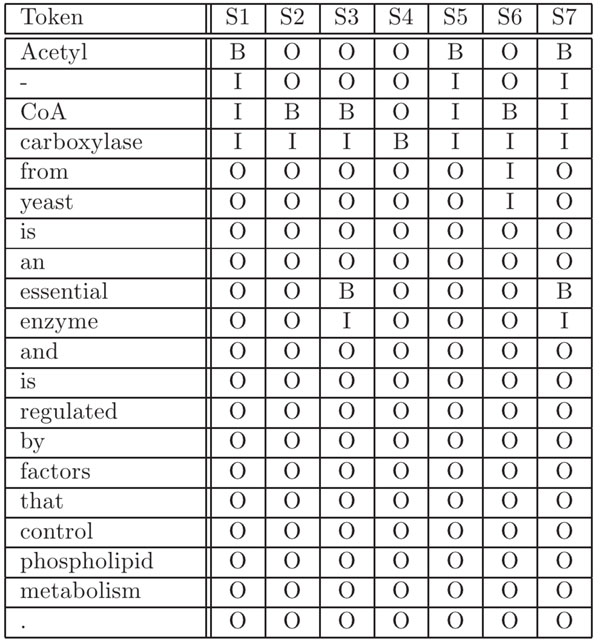
Soft Tagging of Gene Mention Variants

In this case, “Acetyl-CoA carboxylase” is the only gene mention tagging acceptable by bio-curators. Figure 2 shows some of the tagging solutions by a CRF model for this example. The sentence will first be converted into a list of tokens using fine-grained tokenization by breaking any non-alphabet/digit characters. Then a CRF model will label each token using IBO2 representation. Usually, only the most probable labeling will be selected as the solution. But to maximize recall, we let each model generate the top 20 most probable solutions. Figure 2 shows seven of them. These solutions were inferred by the Viterbi “Max-(Product)-Lattice” algorithm implemented in Mallet [20] based on the given CRF model parameters. Since we have forward parsing and backward parsing models, combining their solutions gives 40 solutions for each sentence. We computed union of these solutions for the BC II GM corpus and evaluated the result, which showed that recall of such a tagger was 0.9826 with a precision of 0.1902. That is, this result comprises of nearly all true-positives of the corpus.

These solutions tend to overlap and differ in their boundaries. Decoding the IBO2 representation into human-readable text for the example solutions in Figure 2, we obtain five non-redundant gene mentions:

• “Acetyl-CoA carboxylase”,

• “CoA carboxylase”,

• “essential enzyme”,

• “carboxylase”,

• “CoA carboxylase from yeast”

These gene mentions can be regarded as *variants* of the true positive “Acetyl-CoA carboxylase.” As described above, we expected that one of the variants could be a true-positive. But instead of committing to a single *hard* choice of the boundaries, we make *soft* choices by tagging a set of overlapping variants with different boundaries. With these variants, we can avoid putting efforts into manually creating rules to remove descriptive words preceding or following a gene mention to match gene names in Entrez Gene database, such as {human, rat, mouse, gene, protein, mRNA, cDNA, oncogene}.

However, these variants will overwhelm the GN performance by abundant false positives. There is a need to evaluate the quality of mentions to set priority among variants. Thus, we developed the GME module to estimate a confidence score of each variant. It helps us to remove most false positives without harming the high recall from combined solutions. Tagging overlapping gene and gene product mentions with confidence scores is what we refer to as *soft tagging.* The details of confidence score estimation will be described in next section.

We also used BIOADI [18] to filter out false positives. BIOADI can automatically identify all pairs of abbreviation and its long form definition in an article. If a long form is not tagged in the sentence but its abbreviation is tagged, all appearances of that abbreviation in the article will be marked as invalid and will be eliminated from the result of the GMT module.

For example, “NAA” is a potential gene variant tagged in the following text:

…metabolites in 5 patients: N-acetyl-aspartate (NAA), creatine…

“NAA” is identified by BIOADI as the abbreviation of “N-acetyl-aspartate.” Suppose that a gene mention tagger mistakenly identifies “NAA” as a gene mention. However, from the linkage of “NAA” and “N-acetyl-aspartate,” we can infer that NAA may not a valid gene mention. Therefore, all “NAA” tagged in the sentences of the same article will be considered false positives and eliminated from the output. Table 1 shows the impact of the application of BIOADI to the GN task, evaluated on the training corpus of the BC III GN task. The result shows that it mainly contributed to the precision.

**Table 1:** GN Performance with or without BIOADI on the BioCreative III GN training corpus

Setting	Precision	Recall	F-score	TAP-5	TAP-10	TAP-20
Without BIOADI	0.6248	0.5961	0.6101	0.3252	0.4117	0.4117
With BIOADI	0.6316	0.5911	0.6107	0.3092	0.4190	0.4190

### Soft tagging: gene mention evaluation module

The GME module assigns a confidence score to each of the variants tagged *softly* by the GMT module in the corpus to complete the soft tagging process. We did not use scores from the CRF model as our previous work [14] for this purpose because CRF scores are for the labeling of a whole sentence, where there could be multiple gene mentions. But here we want scores for variants of gene mentions. Ideally, these scores of variants of overlapping gene mentions should reflect the probability that a variant is a true positive.

#### Training data preparation

We used a machine learning method to evaluate and score each variant extracted by the GMT module. The training data was extracted from the BC II GM training corpus (15,000 sentences) where 10,000 sentences were used to train a new CRF model and the remaining 5,000 sentences for testing. The CRF models tagged 90,398 unique instances (variants) which were annotated as either bio-curator approved gene mention or not. The annotation is available from the BioCreative II GM training corpus, which includes manually identified gene mentions and acceptable alternatives (variants) by bio-curators. For instance, {“amniotic fluid alpha fetoprotein”, “fluid alpha fetoprotein”, “alpha fetoprotein”, “alpha” } are four variants of “amniotic fluid alpha fetoprotein”, but only “amniotic fluid alpha fetoprotein” and “alpha fetoprotein” are bio-curator acceptable variants. These variants are annotated as positive examples while others are negatives. We therefore created a new corpus of 23,533 positive and 66,865 negative examples to train machine learning models.

#### Feature design

**Figure 3:**
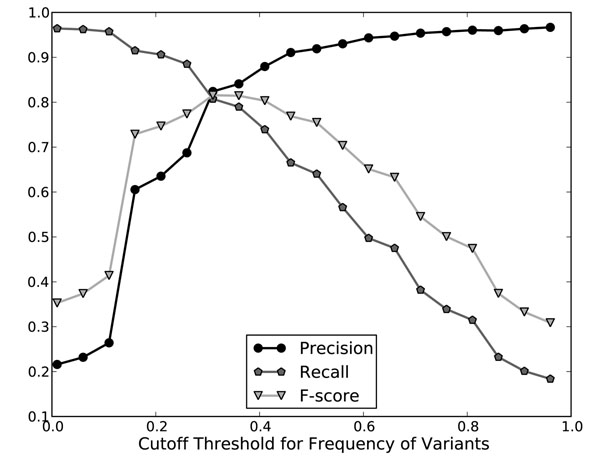
Frequency versus GM Performance

We designed a set of features characterizing the distinction between positive and negative examples and divided them into four types as follows:

• Frequency of Variant: The frequency is a measure of how frequent a variant occurs in the set of combined 40 solutions. For example, consider the set of solutions shown in Figure 2. “Acetyl-CoA carboxylase” is tagged three times, “CoA Carboxylase” and “essential enzyme” twice, and all others once. These counts are then normalized as their frequncy. Figure 3 demonstrated positive correlation between frequency and precision when tested on the BC II GM training corpus, suggesting that a variant with a high frequency is more likely to be a true positive.

• Morphological Features: This type of features describes variants’ morphology to differentiate between common words and gene mentions listed as:

– Has uppercase/lowercase/digit

– Start with uppercase/lowercase/digit

– End with uppercase/lowercase/digit

For example, “IL2R” is an abbreviation of “interleukin 2 receptor.” It starts with an uppercase “I” and ends with an uppercase “R.” Starting and ending with uppercase letters is rare among common English words.

• Content Features: Content tokens are tokens of gene variants. For example, “alkaline” and “phosphatases” are the content tokens of “alkaline phosphatases.” The content tokens of a variant is the most informative to reveal its identity. For example, the word “gene” is one of the tokens in “beta-galactosidase reporter gene”. The token “gene” is a good indicator that “beta-galactosidase reporter gene” is highly likely to be a gene mention. Before extracting this type of features, we normalized each variant to lowercase letters and removed all punctuations, then tokenized it into a list of tokens.

• Contextual Features: The contextual background is also valuable. We defined two tokens preceding and following gene variants as context tokens. For example, the contextual background of “cat” from a passage “…with the cat gene of the S. aureus… ” clearly shows that “cat” is a gene rather than an animal.

From 90,398 training examples, we extracted 385,678 features. Most of them are binary features. Only the frequency of variant is continuous ranging from 0 to 1.

#### Model training and evaluation

We chose the logistic regression model for gene mention evaluation to take advantage of its continuous output between 0 and 1. We regarded the output value as a confidence score to indicate the importance of each variant. By using the features described above, we evaluate the performance of the model by 5-fold cross-validation on the training examples. It achieved an average accuracy of 0.9220 with a standard deviation of 0.0207.

**Figure 4:**
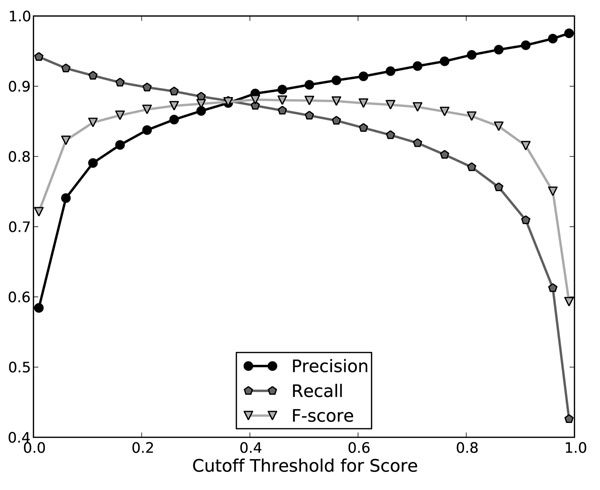
Cutoff Threshold versus GM Performance

We then trained a model with all available examples and regarded it as a scoring function for each variant. To evaluate its efficacy, we tested it on the BioCreative II GM test corpus. By setting different score cutoff thresholds, we can measure the changes of performance related to the thresholds. When tested on the GM test corpus, we designed a recursive algorithm to select exactly one with the maximal score from overlapping variants. Figure 4 shows the performance with different thresholds. The result shows that the precision keeps growing as the threshold increases, while the recall declines. The number of false positives drops from 1210 with no threshold to 627 with threshold = 0.5.

The score helps determining the boundaries of variants, which is crucial to dictionary matching. For example, there are two variants of gene mentions in “human Na,K-ATPase beta subunit,” with their scores: {“human Na,K-ATPase beta”, 0.9957} and {“human Na,K-ATPase”, 0.9316}. Without “beta,” it would be impossible to retrieve the correct gene ID.

In our GN system, we did not select the best variants but only use the threshold for filtering. For the BC 3 GN task, we used 0.2 as the threshold.

### Species designation module

One of the major causes of gene name ambiguity is sharing of gene names of different species. Otherwise, ambiguous names are rare for genes of a single species. The ambiguity mostly comes from homology, gene family, and overlaps and evolving of nomenclature standards. Chen et al. [19] reported a study showing that only 0.02% official symbols were ambiguous within the organisms but for 21 organisms combined, 14.2% of symbols were ambiguous. The dictionary provided for the BC II GN task contains a large collection of unofficial synonyms of gene names for human. Among 177,200 distinct names for 32,975 gene entities, only 4,267, or 2.408%, are ambiguous, confirming that even if unofficial aliases are considered, ambiguous names are relatively rare for human genes. Consequently, a better strategy of GN is to designate species before assigning a gene ID for a candidate gene mention.

The species designation module is to designate each variant a NCBI taxonomy database [21] standard identifier (TaxID). We built a module based on dictionary matching and three heuristic designation rules to achieve the goal. By merging dictionaries of the NCBI taxonomy database and LINNAEUS [22], which contains dictionary packs for cell lines-species and genera-species reference, we established a dictionary of 5,770 entries of species names (included in Entrez Gene records). Each entry is linked to a specific TaxID. In this dictionary, each TaxID has a median of 10 variants of species names ranging from 2 to 35,022 (human).

To assign species to variants, we used three rules as described in [23]. The first rule (Rule 1) is to assign TaxID of the species mentioned within or preceding the variant (including prefix characters, such as {h, y, m, r, d} indicate {human, yeast, mouse, rat, fruit fly}, respectively). The second rule (Rule 2) is to assign TaxID located in the same sentence. Lastly, the third rule (Rule 3) is to assign TaxID of the species mentioned most often in the entire article. With Rule 3, we can assure that all variants will be assigned with one TaxID.

### Gene normalization module

The GN module that we built is to assign a set of overlapping gene mention variants to a specific identifier (GeneID) of Entrez Gene records [24]. Previous works in GN emphasized heavily on disambiguation of multiple matches of a gene mention against a dictionary. However, previous works mostly tried to solve single-species GN from abstracts, while gene name ambiguity in a single species is rare. Then where did all the ambiguous matches come from? Our answer is that this is because the coverage of dictionaries is not sufficient to cover all gene name aliases and their spelling variants. To deal with this, many GN systems used approximate string matching and created many “artificial ambiguities.”

One of our solutions to deal with this problem is to retain all overlapping variants with confidence scores higher than a threshold by soft tagging. The idea is to increase the chance to find a match in the dictionary. Our solutions also include to designate species first and to construct a high coverage dictionary. However, due to time and resource constraints, it was prohibitive for us to construct a high coverage dictionary before the submission deadline. Instead, we extended the indexing and retrieval functions of a document retrieval system for dictionary matching as our current solution. The resulting module is composed of two phases: retrieval phase and resolving phase.

#### Retrieval phase

We designed the retrieval phase to speed up the retrieval of the most relevant gene entries and to narrow down the search space to a small fraction of gene entries to reduce the expensive computation in the resolving phase. We used Apache Lucene [25], a high-performance and full-featured text search engine library, to index the entire Entrez Gene entries and to retrieve the corresponding gene entries of gene mention variants.

We obtained gene names from the columns of {Symbol, Synonyms, dbXrefs, description, Symbol from nomenclature authority, Full name from nomenclature authority, Other designations} of gene_info.gz [26]. We created an index for each gene name with its sources TaxID, GeneID and column header and then stored it in Lucene. A gene name will be listed with its sources as in this example:

(“TaxID:9606”, “GeneID:3552”, “IL1-ALPHA”, “Synonyms”).

Before tokenization performed by Lucene for indexing, we passed the text of the name through a preprocessing step of five rules given in Table 2 to cover more spelling variants and changed all uppercase letters into lowercase letters to ignore case sensitivity. Besides, {1, 2, 3, …} and {I, II, III, …}, as well as {alpha, beta, gamma, …} and {a, b, g, …}, were regarded as interchangeable. Compiling a query consists of two steps:

**Table 2:** Rules for Tokenization before Lucene Indexing

Rule	Regular Expression	Replacement
1	([A-Z]{2,})([a-z]{2,})	$1_⌴_$2
2	([a-z]{2,})([A-Z]{2,})	$1_⌴_$2
3	[\w\_&&[^\.]]	_⌴_
4	([\d\.]+)	_⌴_$1_⌴_
5	\s+	_⌴_

_⌴_ is used to represent a character space.

1. We transform gene variants with the rules given in Table 2 to obtain a normalized (extended text) for gene name search. In this example, the gene name “IL1-ALPHA” will be transformed into the follows: i1_⌴_(?i:1|i)_⌴_(?i:a|alpha)

2. We use the taxonomy id obtained from the species designation module as the second criterion for TaxID search. In this case, the TaxID will be 9606 (*Homo sapiens*)*.*

To retrieve the most relevant entries against a given set of variants, we compiled a query with each variant text and its TaxID to search the indexed entries in Lucene. We regarded the top 50 search results returned by Lucene as the candidates. For example, the search results listed in Table 3 are Lucene’s search results with regard to the query (TaxID:9606, “IL-1 alpha”). Then a regular expression pattern derived from the query text were used to filter out the results whose names failed to match the pattern before the search results were passed to the next phase. This process is necessary to remove partial matches returned by Lucene. In this case, the query text is “IL-1 alpha” and the query results are given in Table 3. The entries with the gene names of {IL1A, IL1ALPHA, IL-1A, IL-1 alpha} will be retained because they match the regular expression pattern of “IL-1 alpha”.

**Table 3:** Example of Top 20 Names Retrieved by Lucene in the GN Retrieval Phase

TaxID	GeneID	Name	Column Header	Lucene Score
9606	3552	ILIA	Synonyms	1.0000
9606	3552	IL1ALPHA	Synonyms	1.0000
9606	3552	IL-1A	Synonyms	1.0000
9606	3552	IL1A	AuthorizedSymbol	1.0000
9606	3552	IL1-ALPHA	Synonyms	1.0000
9606	3552	IL1A	Symbol	1.0000
9606	3552	IL-1 alpha	OtherDesignations	1.0000
9606	3554	IL-1R-alpha	Synonyms	1.0000
9606	26525	IL1F5 (Canonical product IL-1F5a)	OtherDesignations	0.7459
9606	27177	IL1F8 (Canonical product IL-1F8a)	OtherDesignations	0.7459
9606	84639	IL-1F10 (canonical form IL-1F10a)	OtherDesignations	0.7459
9606	3552	IL1	Synonyms	0.6417
9606	3553	IL1	Synonyms	0.6417
9606	3553	IL-1	Synonyms	0.6417

Since the GME module retains all overlapping variants unless their confidence scores are below the threshold, for a set of overlapping variants at most a Entrez Gene ID should be returned in the final output. Usually different variants will match the same Entrez Gene ID. But still in some cases, they may match different Entrez Gene IDs. For example, “IL-1 alpha” and “IL-1” are two overlapping variants that match two distinct Entrez Gene IDs. To determine a correct Entrez Gene ID, our approach is to choose the match of the longest variant. This is a simple yet reasonable approach to disambiguation.

#### Resolving phase

The retrieval phase produces a list of entries as shown in Table 4. If there is only one unique GeneID in the list, we assigned the GeneID to the given variant. In this example, “IL-1 alpha” will be assigned to GeneID:3552. If more than one unique GeneID in the list, we used a heuristic approach to GeneID assignment: assign the GeneID of an entry which was extracted from the columns {Symbol, Symbol from nomenclature authority, full name from nomenclature authority} in Entrez Gene Records. If none satisfies this prerequisite, we will ignore this list of entries. We used the score of each variant assigned by the gene mention evaluation module as the final score as the GN output.

**Table 4:** Example of Matched Names in the GN Resolving Phase

TaxID	GeneID	Name	Column Header	Lucene Score
9606	3552	IL1A	Synonyms	1.0000
9606	3552	IL1ALPHA	Synonyms	1.0000
9606	3552	IL-1A	Synonyms	1.0000
9606	3552	IL1A	AuthorizedSymbol	1.0000
9606	3552	IL1-ALPHA	Synonyms	1.0000
9606	3552	IL1A	Symbol	1.0000
9606	3552	IL-1 alpha	OtherDesignations	1.0000

## Results and discussion

### Performance of soft tagging

**Figure 5:**
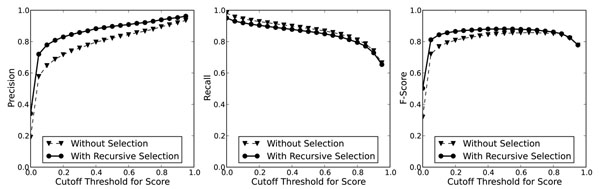
Cutoff Threshold versus Performance on the BioCreative II GM test corpus

Soft tagging is accomplished by the gene mention tagging module and gene mention evaluation module in our gene normalization system. Advantages of soft tagging include: First, we can use the confidence scores to adjust the trade-off between recall and precision by setting a cutoff threshold to remove variants below a threshold. Second, the scores of variants are useful to set priority when we are required to make a choice among them. To analyze the performance of soft tagging as a gene mention tagger, we tested it on the BioCreative II test corpus. Each variant has a confidence score estimated by the GME module. At first, we did not do any selection of these variants by their scores. We then tested its gene mention tagging performance with different thresholds. The results are shown in Figure 5. The results show trade-off between precision and recall. When the threshold is set to 0.2, the recall is 0.9281 and precision is 0.7168, a 6 percent drop in recall from zero threshold but a 50 percent gain in precision. Compared to our previous work [17], the new GM module with the best F-score 0.8576 performs one percent lower than the best GM F-score of our submission to the BC II GM task, which achieved 0.8683 F-score with a precision of 0.8930 and a recall of 0.8449. This is mostly because overlapping tagging solutions contain only at most one true positive but the others are all false positives. The precision will therefore suffer much because of these false positives. To alleviate this type of error, we designed a recursive algorithm to select variants with the highest score within a passage of a text. Thus only variants with the highest score will be tagged as the GM results. The performance with this selection is reported in Figure 5. The F-score was improved to 0.8807 with a precision of 0.8972 and a recall of 0.8648 with 0.5 threshold. This is 1.2 percent higher than our previous work. Compared to its “without-selection” counterpart, it accomplished nearly 3 percent of improvement in F-score from 0.8522 to 0.8807. Consequently, soft tagging with estimated confidence scores can improve the performance of gene mention tagging.

### Evaluation of species designation module

We used three heuristic rules to designate TaxID for each variant. To analyze their impact on gene normalization, we tested their combinations on the BioCreative III Training corpus of 32 full-annotated articles, and regarded Rule 1 as the baseline. The results are listing in Table 5. The use of Rule 1 achieved a F-score of 0.3434, with the highest precision of 0.7432 among all tests. Recall that Rule 1 is the most intuitive rule to designate a species to a variant when a species preceding the variant. Rule 1+2 achieved a 7 percent improvement in the recall, but lost 5 percent in the precision, suggesting that designating species co-located with variants in the same sentence provides modest contribution when Rule 1 is not applicable. Remarkably, Rule 1+2+3 boosts the recall by about 20 percent compared to the performance of Rule 1+2 and Rule 3 only turns out to perform better than both Rule 1 and Rule 1+2. In fact, Rule 3 only even outperforms Rule 1+2+3 in TAP-5 even though its F-score is worse. This may be due to the fact that the number of output gene IDs using Rule 3 only is larger than that using Rule 1+2+3 and TAP-k favors a long list of output. Performance of Rule 3 shows that analyzing text is required to designate species for a large proportion of variants. A reasonable explanation is that papers usually focus on a small number of organisms (e.g., model organisms in experiments for a study of a human disease).

**Table 5:** GN Performance versus Species Designation Rules

Rule(s)	Precision	Recall	F-score	TAP-5	TAP-10	TAP-20
1	0.7432	0.2233	0.3434	0.2983	0.2983	0.2983
1+2	0.6980	0.2923	0.4120	0.3353	0.3353	0.3353
3	0.6360	0.5107	0.5665	0.3795	0.3926	0.3926
1+2+3	0.6316	0.5911	0.6107	0.3092	0.4190	0.4190

### Analysis of gene normalization module

We submitted 3 runs for the BioCreative III GN task evaluation. As we know, the Entrez Gene database is frequently updated. Run 1 and Run 2 used two different versions of Entrez Gene downloaded in May 2010 and July 2010, respectively. Run 3 is the union of Run 1 and Run 2. The most remarkable difference between the versions of May and July is that all records of TaxID:4932 (Saccharomyces cerevisiae) were moved to TaxID:559292 (Saccharomyces cerevisiae S288c). This change caused a conflict with our taxonomy dictionary, an integration of LINNEAUS and NCBI taxonomy database downloaded in May 2010. All variants with TaxID:4932 cannot be correctly designated to Entrez gene entries, and must be redirected to TaxID:559292 for the version downloaded in July 2010.

To let participants evaluate the performance of these submitted runs, the BioCreative organizers released four annotations of the BioCreative III GN test corpus with TAP-k [27] evaluation scripts version 1.3. One of them is annotated by professional bio-curators and the others are annotated automatically by team submissions using the EM algorithm [12]. Table 6 reports the performance of our submitted runs on these annotations. We observed that there is no significant performance difference in F-scores and TAP scores (*k* =5, 10 and 20) between Run 1 and Run 2 on these annotations. Next, we found that the scores for the silver annotations are usually double of those for the gold annotation of 50 articles of the BioCreative III GN test corpus, selected from a total of 507 articles. Our GN system accomplished high precisions for the silver annotations, suggesting that our results were consistent with other participants’ results.

**Table 6:** Performance of Submitted Runs on the BioCreative III GN test corpus

Annotation	Run	Precision	Recall	F-score	TAP-5	TAP-10	TAP-20
	R1	0.4494	0.2316	0.3056	0.2137	0.2509	0.2509
test50.gold	R2	0.4289	0.2352	0.3038	0.2086	0.2483	0.2483
	R3	0.4237	0.2364	0.3034	0.2099	0.2495	0.2495

	R1	0.8801	0.4136	0.5627	0.3820	0.3820	0.3820
test50.silver	R2	0.8632	0.4316	0.5755	0.3855	0.3855	0.3855
	R3	0.8570	0.4360	0.5780	0.3890	0.3890	0.3890

	R1	0.8433	0.4327	0.5720	0.4540	0.4540	0.4540
test507.silver1	R2	0.8272	0.4377	0.5724	0.4536	0.4536	0.4536
	R3	0.8233	0.4427	0.5758	0.4577	0.4577	0.4577

	R1	0.9185	0.4743	0.6256	0.4873	0.4873	0.4873
test507.silver2	R2	0.9048	0.4818	0.6287	0.4871	0.4871	0.4871
	R3	0.9009	0.4875	0.6326	0.4916	0.4916	0.4916

test50.gold: human annotation for the 50 articles

test50.silver: pooled team submissions for the same 50 articles using the EM algorithm

test507.silver1: human annotation for the 50 articles + pooled team results for the remaining 457 articles

test507.silver2: pooled team submissions for all the 507 articles by the EM algorithm

In the BioCreative III GN test evaluation, our Run 3 was ranked the first for the 50 articles using the silver standard annotations in TAP (*k*=5) among 37 submitted runs from 14 teams and never ranked lower than the third for any combination of annotations and *k* settings. We observed that the TAP-k scores are usually the same in our system. We suspected that it is because our system produced a short list of records for an article to have less then *k* false positives to appear and caused the TAP scores to freeze at the value of TAP-5. Table 7 shows the min, median and max lengths of the output gene ID lists from our GN system and the min, median and max number of false positives, as well as the percentage of the number of missed genes (false-negatives). Compared to “Truth,” the standard annotations provided by the organizers, apparently our output lists are short but our system produced remarkably few false positives especially for silver standards.

**Table 7:** Analysis of Submitted Runs on the BioCreative III GN test corpus

Annotation	Run	GN records			False-Positives			False-Negatives
		
		Min	Median	Max	Min	Median	Max	Total (%)
	Truth	1	19	375				
	
test50.gold	R1	1	18	80	1	9	35	1283 (76.87)
	R2	1	19	82	1	10	44	1277 (76.51)
	R3	1	19	84	1	10	45	1275 (76.39)

	Truth	1	36	219				
	
test50.silver	R1	1	18	80	1	2	14	1072 (58.64)
	R2	1	19	82	1	3	20	1039 (56.83)
	R3	1	19	84	1	3	20	1031 (56.40)

	Truth	1	12	375				
	
test507.silver1	R1	1	9	80	1	1	35	5342 (56.96)
	R2	1	9	82	1	1	44	5296 (56.47)
	R3	1	10	84	1	2	45	5249 (55.97)

	Truth	1	11	219				
	
test507.silver2	R1	1	9	80	1	1	14	4927 (52.84)
	R2	1	9	82	1	1	20	4858 (52.10)
	R3	1	10	84	1	1	20	4805 (51.53)

### Future work

Our future work is to improve the GN system in a number of ways. First, our current system is unable to extract GN records from figures [28] and tables. Substantial gene information may appear in captions of figures and tables. Missing gene mentions in figures and tables contributed to the low recall of our GN system. Second, it is still challenging to match Entrez Gene records with tagged gene mentions without accurate species designation. It is quite likely that improvement in species designation will result in substantial gain in performance. Finally, we plan to develop a trainable model for the entire GN process so that the system will improve its performance with more training examples. Important progress in biomedical literature mining may be accomplished by the development of models that learn progressively throughout the task [3,29], that will create a positive feedback loop [30]. Currently, our gene normalization module is not trainable. It is possible to develop a machine learning method to filter results of dictionary matching and to resolve disambiguation if necessary [31].

## Conclusions

The key feature of our GN system is that we applied a novel soft tagging approach that produces sets of high confidence overlapping gene mention variants to maximize the chance of precise match in a dictionary which is similar to Baumgartner et al. [10] while minimizing artificial ambiguities due to the use of approximate string matching to compensate low coverage of dictionaries. Disambiguation by considering contextual information is still important to a practical GN system. The lack of use of any contextual information in the disambiguation in our GN system is mostly due to time and resource constraints to meet the submission deadline. Despite the lack of clever disambiguation, we show that soft gene mention tagging is sufficient to produce competitive gene normalization results in a cross-species and full-text setting. This evaluation suggests that large improvement is required to make automatic gene normalization practical. However, given this encouraging result, we are confident that by combining soft tagging with trainable species designation and context-based disambiguation, the resulting system will perform much better to actually meet the needs of bio-curation.

## Competing interests

The authors declare that they have no competing interests.

## Authors contributions

CJK and MHTL developed methods and drafted the manuscript. CNH was responsible for all aspects of the project and helped revise the manuscript.
